# Switching Bond: Generation of New Antimicrobial Peptides via the Incorporation of an Intramolecular Isopeptide Bond

**DOI:** 10.1021/acsinfecdis.1c00037

**Published:** 2021-05-27

**Authors:** Naiem Ahmad Wani, Daniel Ben Hur, Gal Kapach, Elad Stolovicki, Etai Rotem, Yechiel Shai

**Affiliations:** Department of Biomolecular Sciences, Weizmann Institute of Science, Rehovot 76100, Israel

**Keywords:** antimicrobial peptide, isopeptide bond, pexiganan, Gram-positive bacteria, Gram-negative bacteria

## Abstract

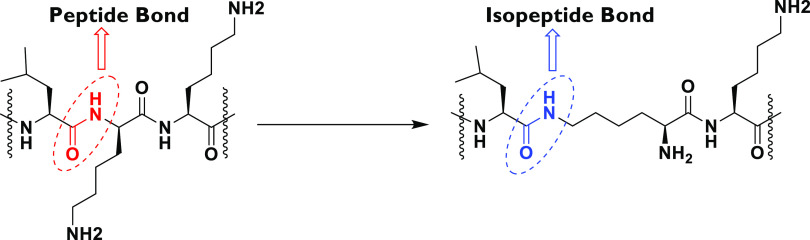

Antimicrobial peptides (AMPs), which can be modified to kill a broad spectrum of microoganisms or a specific microorganism, are considered as promising alternatives to combat the rapidly widespread, resistant bacterial infections. However, there are still several obstacles to overcome. These include toxicity, stability, and the ability to interfere with the immune response and bacterial resistance. To overcome these challenges, we herein replaced the regular peptide bonds with isopeptide bonds to produce new AMPs based on the well-known synthetic peptides Amp1L and MSI-78 (pexiganan). Two new peptides Amp1EP and MSIEP were generated while retaining properties such as size, sequence, charge, and molecular weight. These new peptides have reduced toxicity toward murine macrophage (RAW 264.7) cells, human monocytic (THP-1) cells, and human red blood cells (hRBCs) and enhanced the stability toward proteolytic degradation. Importantly, the new peptides do not repress the pro-inflammatory cytokine and hence should not modulate the immune response. Structurally, the new peptides, Amp1EP and MSIEP, have a structure of random coils in contrast to the helical structures of the parental peptides as revealed by circular dichroism (CD) analysis. Their mode of action, assessed by flow cytometry, includes permeabilization of the bacterial membrane. Overall, we present here a new approach to modulate AMPs to develop antimicrobial peptides for future therapeutic purposes.

Antimicrobial peptides (AMPs) are innate immunity polypeptides serving as the first line of defense against pathogens in all species of life.^1−5^ Bacterial infections caused by multidrug-resistant bacteria are rapidly spreading and represent a major problem worldwide. Antibiotics with new mechanisms of action are urgently required to combat the growing health threat posed by resistant pathogenic microorganisms.^6,7^ Thus, the development of antibiotics that will overcome bacterial resistance is one of the most important and difficult challenges in microbiology. Due to the high potency of AMPs and their various modes of action, they are considered to be a promising alternative to conventional antibiotics.^8−13^ A particular native AMP can be easily modified to create new peptidic or nonpeptidic analogs, many of which preserve antimicrobial activity. In addition, they gain or lose other activities, such as hemolysis, cell toxicity, immunomodulation, endotoxin neutralization, and others.^14,15^ Most AMPs are believed to exert their antimicrobial activity partially through membrane permeation and disruption.^16−18^ However, despite the advantages described above, antimicrobial peptides are quite poorly used in clinical practice because of several challenges, such as hemolytic and/or cytotoxic activity and susceptibility to degradation by host proteases.^3,19,20^ New AMPs with potent antimicrobial activity, resistance to proteolysis, and low toxicity have been designed by several approaches.^21−23^ For example, modifications performed on native peptides, which include peptidomimetics, truncation of native peptide, and termini modifications, demonstrated beneficial effects on activity and toxicity.^22,24−27^ Some AMPs such as Iseganan, Neuprex, Surotomycin, XMP-629, and MSI-78 commercially known as pexiganan, which is an analog of magainin-2,^10,28−30^ have had limited success in clinical settings primarily due to nonspecific cell toxicity and poor biological stability and hemolytic activity.^19,31−34^ The quest to produce AMPs with an effective antimicrobial activity, low toxicity, low ability to interfere with the immune response, and high stability is a major obstacle to unleash the potential of AMPs. Towards this goal, we synthesized and investigated two new antimicrobial peptides named Amp1EP and MSIEP; both relay on previously discovered AMPs, Amp1L,^35^ and MSI-78.^36^ Importantly, for the first time, we replaced peptide bonds by amide bond isosteres in the backbone of these peptides while retaining properties such as size, sequence charge, and molecular weight. The activity of these new peptides was tested against a panel of two Gram-negative (*P. aeruginosa* PA01 and *E. coli* K12 parental type) and two Gram-positive (*S. aureus* and *B. subtilis*) bacteria. These peptides showed antimicrobial activity against all the bacteria strains tested. Importantly, the peptides showed reduced toxicity toward human red blood cells (hRBCs), murine macrophage (RAW 264.7) cells, and human monocytic (THP-1) cells as well as enhanced protection from proteolytic degradation. Furthermore, the membrane-penetrating activity of all the peptides using flow cytometry showed compromised membrane integrity in Gram-negative bacteria. Altogether, we present here a new approach to modulate AMPs, while keeping their size, sequence, and hence general mode of action. From a practical point of view, this approach can be extended to develop antimicrobial peptides for future therapeutic purposes with enhanced stability and prolonged activity.

## Results

**Figure 1:**
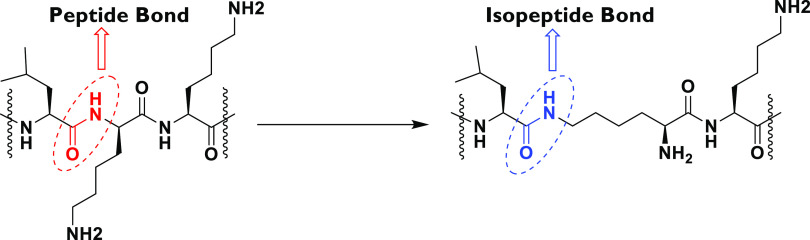
General chemical structure of a peptide bond versus an isopeptide bond.

Previous studies demonstrated that Amp1L and MSI-78 (pexiganan) have a potent antimicrobial activity, but both are quite hemolytic and toxic toward mammalian cells.^24,34,37,38^ To reduce such undesirable properties, two new AMPs, Amp1EP and MSIEP, were synthesized from the parental peptides with an incorporation of three isopeptide bonds while retaining their size, sequence, charge, and molecular weight (Table 1). There are differences in the relative hydrophobicities of the peptides, which are overall low. The general chemical structure of the peptide bond versus an isopeptide bond is shown in Figure 1.

**Table 1:** Designations, Sequences, and Relative Hydrophobicities of Peptides^a^

designation	sequence^b^	net charge	length (amino acids)	mol. wt	relative hydrophobicity^c^ (% AcN)	retention time^d^ (min)
Amp1L	LKLLKKLLKKLLKLL	+7	15	1805	59	24.5
Amp1EP	LKLL**K**KLL**K**KLL**K**LL	+7	15	1805	49.4	19.7
MSI-78	GIGKFLKKAKKFGKAFVKILKK	+9	22	2477	52.6	21.3
MSIEP	GIGKFLK**K**AKKFG**K**AFV**K**ILKK	+9	22	2477	34.4	12.2

a Underlined and bolded lysines (K5, K9, and K13 in the case of Amp1EP and K8, K14, and K18 in the case of MSIEP) are involved in isopeptide bond formation.

b All of the peptides are amidated at their C-termini.

c Relative hydrophobicity is reflected by the percent of acetonitrile at the retention time.

d Reversed-phase HPLC retention time in the C_4_ column by using a gradient of 10–90% acetonitrile in water for 40 min.

### Antibacterial Activity of the Peptides

A panel of four bacteria, including two Gram-negative (*P. aeruginosa*, *E. coli*) and two Gram-positive (*S. aureus*, *B. subtilis*) bacterial strains, was used to test the antibacterial properties of the peptides. The bacteria *P. aeruginosa*, *E. coli*, and *S. aureus* belong to the ESKAPE group of pathogens, which are potential multidrug resistant strains that are majorly involved in various nosocomial infections. Antibacterial activity was measured in terms of minimal inhibitory concentration (MIC), i.e., the lowest concentration of a peptide that fully inhibits bacterial growth. The new peptides Amp1EP and MSIEP are potent against all the bacteria tested. However, the antimicrobial activity of these new peptides was higher than the parental peptides as shown in Table 2.

**Table 2:** Antibacterial Activity of the Peptides

	MIC (μM) of Peptides			
microorganisms	Amp1L	Amp1EP	MSI-78	MSIEP
*P. aeruginosa*	0.78	3.125–6.25	0.78	6.25–12.5
*E. coli*	3.12–6.25	6.25	1.56–3.12	12.5
*S. aureus*	1.56–3.25	1.56–3.25	0.78–1.56	6.25–12.5
*B. subtilis*	6.25–12.5	12.5	1.56	6.25–12.5

### Cytotoxicity of the AMPs

**Figure 2:**
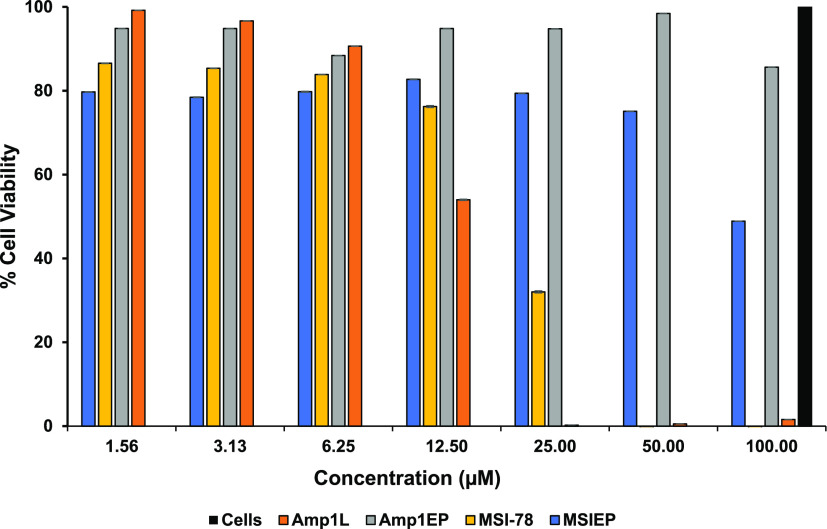
Cytotoxicity of peptides (1.56–100 μM) on RAW 264.7 cells. Cell viability was analyzed and quantified by measuring the absorption at 450 nm. The data is presented as mean percent viability. All data represent mean ± SD from three independent experiments. One-way analysis of variance was used to analyze the data. Results show a statistically significant difference (*F* = 58.83; *p* < 0.001).

**Figure 3:**
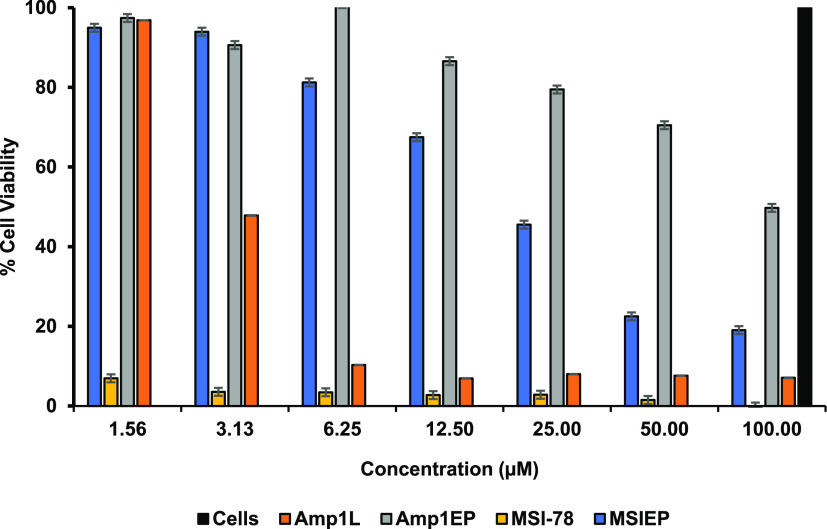
Cytotoxicity of peptides (1.56–100 μM) on human monocytic (THP-1) cells. Cell viability was analyzed and quantified by measuring the absorption at 450 nm. The data is presented as mean percent viability. All data represent mean ± SD from three independent experiments. One-way analysis of variance was used to analyze the data. Results show a statistically significant difference (*F* = 63.73; *p* < 0.001).

Peptide-induced cytotoxicity was assessed against murine macrophage (RAW 264.7) cells and human monocytic (THP-1) cells using the benzenesulfonic acid hydrate and *N*-methyl dibenzopyrazine methyl sulfate (XTT) dye reduction assay. The parental peptides Amp1L and MSI-78 were toxic to RAW 264.7 cells at 25 and 50 μM and THP-1 cells at 6.25 and 1.56 μM (Figures 2 and 3, respectively). It is noteworthy to note that, in the case of RAW 264.7 cells, new peptides Am1EP and MSIEP reduced the cell viability only by 14% and 50% at 100 μM (Figure 2), while as in the case of THP-1 cells, cell viability was dropped by 50% and 20%, respectively, at 100 μM of the highest concentration tested (Figure 3). This may in part be due to low hydrophobicity (Table 1), leading to less bacterial membrane alteration.

### Hemolytic Activity of the Peptides

**Figure 4:**
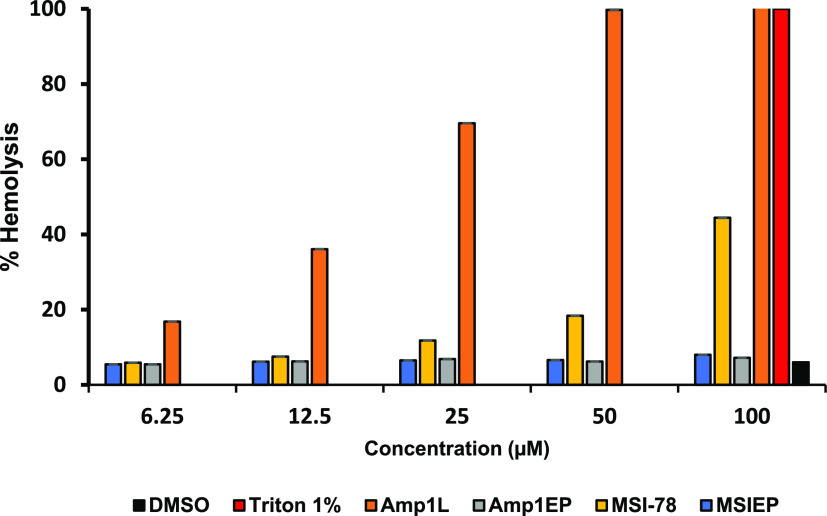
Hemolytic activity of the peptides (6.25–100 μM) on human red blood cells (hRBCs). Untreated cells were used as a negative control, and cells treated with 1% Triton X-100 were used as a positive control. All data represent mean ± SD from three independent experiments performed in duplicate. One-way analysis of variance was used to analyze the data. Results show a statistically significant difference (*F* = 50.63; *p* < 0.001).

The hemolytic activity of the peptides Amp1L, Amp1EP, MSI-78, and MSIEP was tested against human red blood cells (RBCs). The release of hemoglobin (Hb) was measured at OD_450_ nm using a microplate autoreader. As indicated in Figure 4, Amp1EP and MSIEP showed no hemolysis even at the highest concentration tested, i.e., 100 μM, while the parental peptide Amp1L was hemolytic at 6.25 μM, the lowest concentration tested, and MSI-78 showed >20% hemolysis at 50 μM. The reduction in the hemolytic activity is probably due to the reduction in both the hydrophobicity and the α-helical structure of the new peptides.

### Enzymatic Degradation of the Peptides

**Figure 5:**
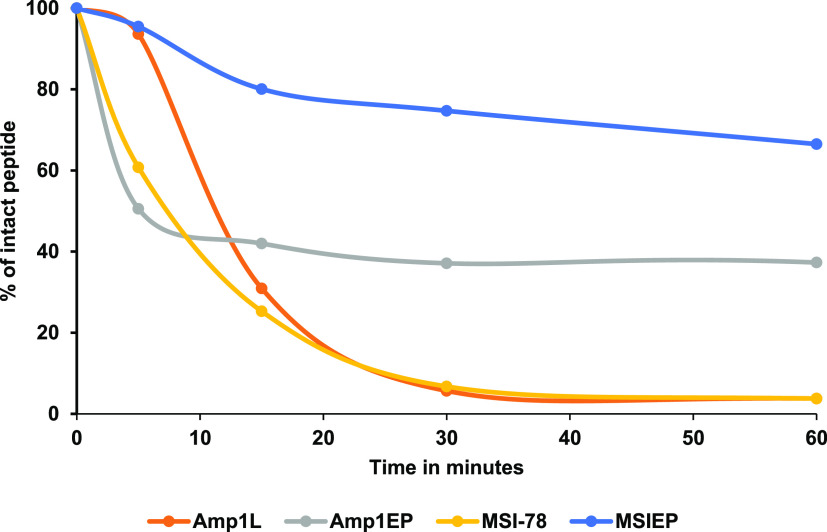
Resistance of peptides to trypsin digestion. Percentages intact of Amp1L, Amp1EP, MSI-78, and MSIEP peptides were determined by reversed-phase HPLC comparative to the peak areas acquired at *t*_0_ (control at 0 min set to 100% for each peak).

In order to overcome the proteolytic instability of peptide based drugs, the resistance of peptides to proteases is an essential prerequisite^33^ because the peptides can be digested by the microbial or host protease and lose activity. Here, we tested the effect of the incorporation of an amide bond isostere on the resistance to trypsin, a serine protease known to cleave peptide bonds mainly at the C-terminal side of the amino acids lysine or arginine. For that purpose, the peptides Amp1EP and MSIEP and their parental peptides Amp1L and MSI-78 were incubated in trypsin and the degradation was quantified by reversed-phase HPLC (Figure 5). After incubation for 1 h, more than 66% of MSIEP and 37% of Amp1EP remained intact. In comparison, MSI-78 and Amp1L were no longer detected by HPLC after 1 h. This result verified the stability of the peptides modified with isopeptide bonds with respect to their counterparts.

### Proteolytic Stability in Human Blood Plasma

The stability of the peptides was assessed by incubating them in blood plasma (20% v/v) rich in proteases thrombin and plasmin for different time periods at 37 °C. Table 3 shows the amount of the remaining peptide following the incubation with plasma at different time points. It is revealed that Amp1EP and MSIEP displayed significantly higher resistance against proteolytic cleavage compared to Amp1L and MSI-78 (intact Amp1EP and MSIEP peptide at ∼85% and 99% in comparison to intact Amp1L and MSI-78 peptide at ∼65% and 81% after a 30 min incubation period, respectively). After 120 and 240 min incubation, the remaining percentages of Amp1L were ∼61% and 39% as compared to 60% and 37% for Amp1EP, while 75% of MSIEP remained intact even after 240 min compared to MSI-78 where the percentage of intact peptide was only 55% (Table 3). Overall, the results demonstrated that Amp1EP displayed higher stability up to the period of 30 min, whereas after 240 min of incubation with plasma, Amp1L and Amp1EP exhibited almost similar stability, while MSIEP was relatively more stable even after 240 min compared to MSI-78.

**Table 3:** Amount of % Remaining Amp1L, Amp1EP, MSI-78, and MSIEP Peptides Assessed by RP-HPLC after Incubation with 20% (v/v) Human Plasma at Different Time Points

	percentage intact peptide			
time (minutes)	Amp1L	Amp1EP	MSI-78	MSIEP
0	100	100	100	100
15	71	85	91	99
30	65	85	81	98
60	62	66	81	76
120	61	60	63	75
240	39	37	55	75

### Structural Characterization of the Peptides

**Figure 6:**
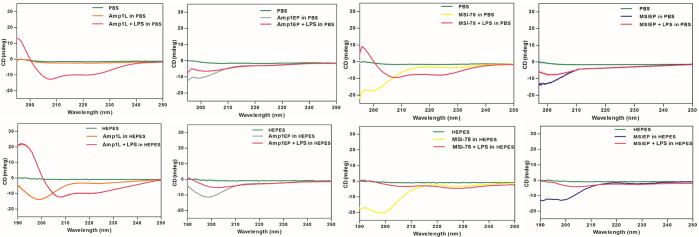
Secondary structures of peptides by circular dichroism (CD). CD spectra of Amp1L, Amp1EP, MSI-78, and MSIEP in PBS, LPS + PBS, HEPES, and LPS + HEPES.

To examine the secondary structure of the peptides, circular dichroism (CD) experiments were performed in PBS and 5 mM HEPES (pH 7.4) to a final concentration of 50 μM peptide. The results revealed that none of these peptides displayed any considerable secondary structures in PBS, while in HEPES, all peptides exhibited random coil conformations (Figure 6). Further, the peptides were tested in the presence of LPS (1:1 ratio, LPS/peptide), and the results revealed that Amp1L and MSI-78 adopted defined helical structures, whereas the corresponding new peptides Amp1EP and MSIEP did not show significant helical structures (Figure 6).

### Investigation of the Permeability of the Bacterial Membrane by Flow Cytometry and Confocal Microscopy Using the SYTOX Green Uptake Assay

**Figure 7:**
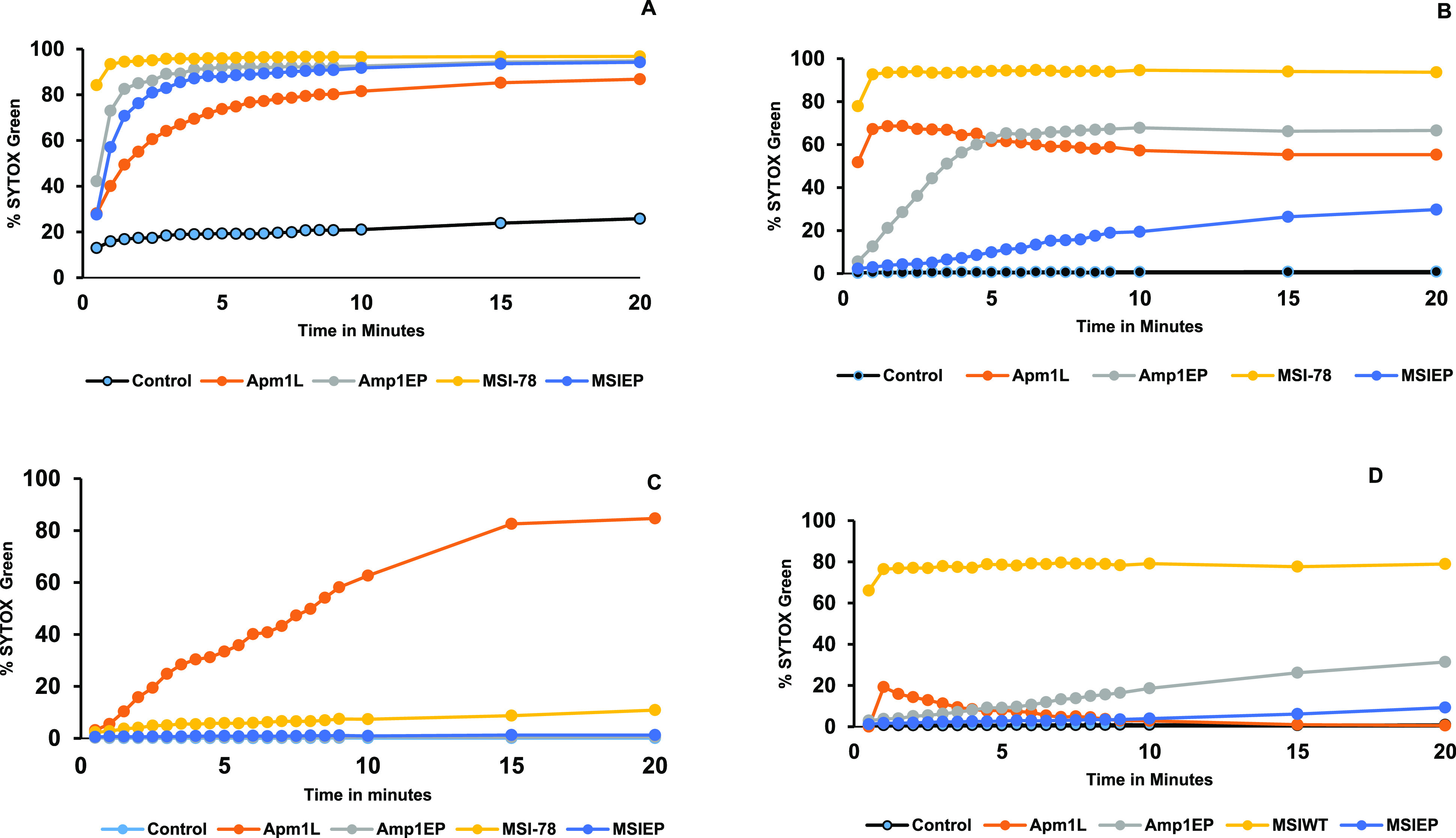
Bacterial membrane permeabilization. (A) *P. aeruginosa*, (B) *E. coli*, (C) *S. aureus*, and (D) *B. subtilis* bacteria using SYTOX green were measured with a flow cytometer in the presence or absence of peptides for 20 min.

**Figure 8:**
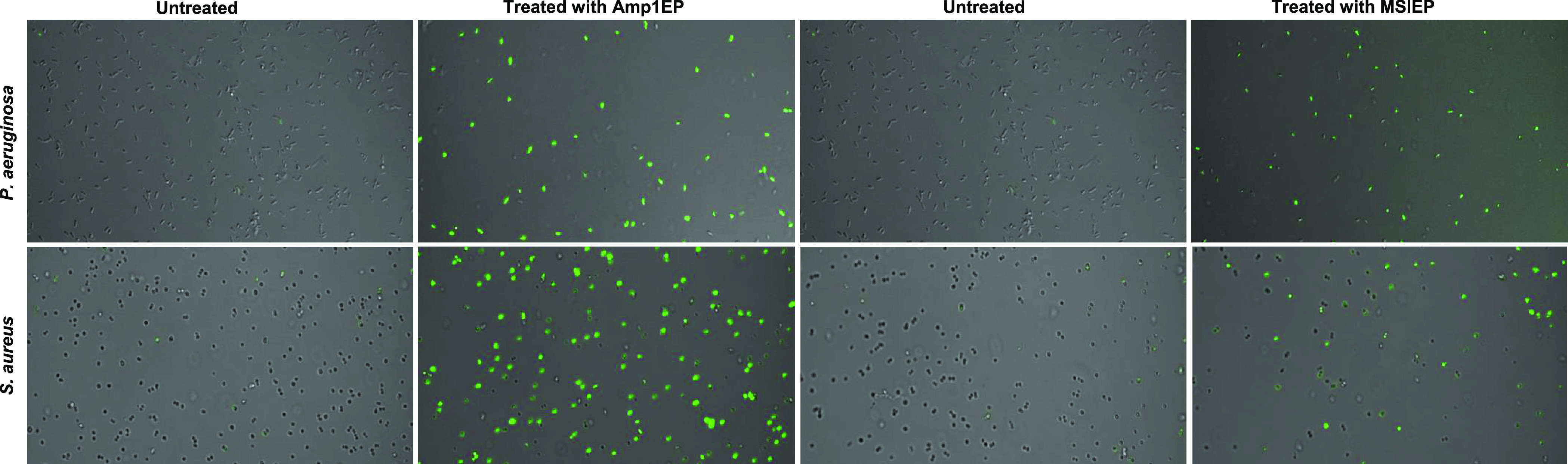
Peptide-induced influx of SYTOX green into *P. aeruginosa* and *S. aureus* bacterial cells permeabilized the cell membrane. After treatment with or without Amp1EP and MSIEP, the membrane permeation of *P. aeruginosa* and *S. aureus* was determined by confocal microscopy.

To evaluate the possible mechanism of action of the AMPs on bacteria, membrane permeability was studied by staining them with the SYTOX green assay. The cationic DNA dye SYTOX green penetrates and labels only the cells with disrupted membranes. Since SYTOX green fluorescence signal increases on binding to DNA, it enabled us to follow the dynamics of the puncturing of the bacterial membrane in real time without an additional washing step. Flow cytometry has the advantages of rapidly acquiring a large number of cells, providing immediate quantitative results and potentially increasing signal-to-noise ratio by filtering out signals from the free DNA in the solution. The peptides at MIC concentrations were added to the bacterial suspension supplemented with SYOX green, and the suspensions were measured in the flow cytometer continuously for 20 min. The rate of dye penetration to the bacteria depends on the bacteria and the peptide used. All the peptides permeabilized the membrane of all the bacteria tested. Among the new peptides, Amp1EP permeabilized the membranes of *P. aeruginosa*, *E. coli*, and *B. subtilis*, showing a marked enhancement in the fluorescence over time in contrast to Amp1L, which showed a slower elevation of fluorescence, whereas Amp1L showed a greater elevation in fluorescence over time in the case of *S. aureus* as compared to Amp1EP. The treatment of peptide MSI-78 showed a sharp increase in the SYTOX green influx in *P. aeruginosa*, *E. coli*, and *B. subtilis*, whereas in case of *S. aureus*, there was a low percentage of SYTOX green influx. However, in case of MSIEP, there was a moderate effect on *P. aeruginosa*, *E. coli*, and *B. subtilis* and no effect on *S. aureus* at all. The fractions of penetrated cells over time are presented in Figure 7, and the results at the end of the experiment, after 20 min, are summarized in Table 4. For further understanding of the effect of the peptides on the bacteria membrane, we investigated the process using a confocal microscope. We treated *P. aeruginosa* and *S. aureus* with the new peptides Amp1EP and MSIEP and observed the penetration of SYTOX green to the cells. Both new peptide treatments increased the green fluorescence signal from the target bacteria *P. aeruginosa* and *S. aureus* as compared with the control, untreated cells Figure 8.

**Table 4:** Percentage of Bacterial Membrane Permeabilization of Peptides

	percentage of membrane permeable bacteria after 20 min				
	peptides				
	control	Amp1L	Amp1EP	MSI-78	MSIEP
*P. aeruginosa*	0	86.8	94.87	96.77	94.24
*E. coli*	0	55.35	66.62	93.68	29.78
*S. aureus*	0.11	84.67	0.56	10.85	1.25
*B. subtilis*	0	0.55	31.38	78.92	9.27

### Cytokines Release Assay

**Figure 9:**
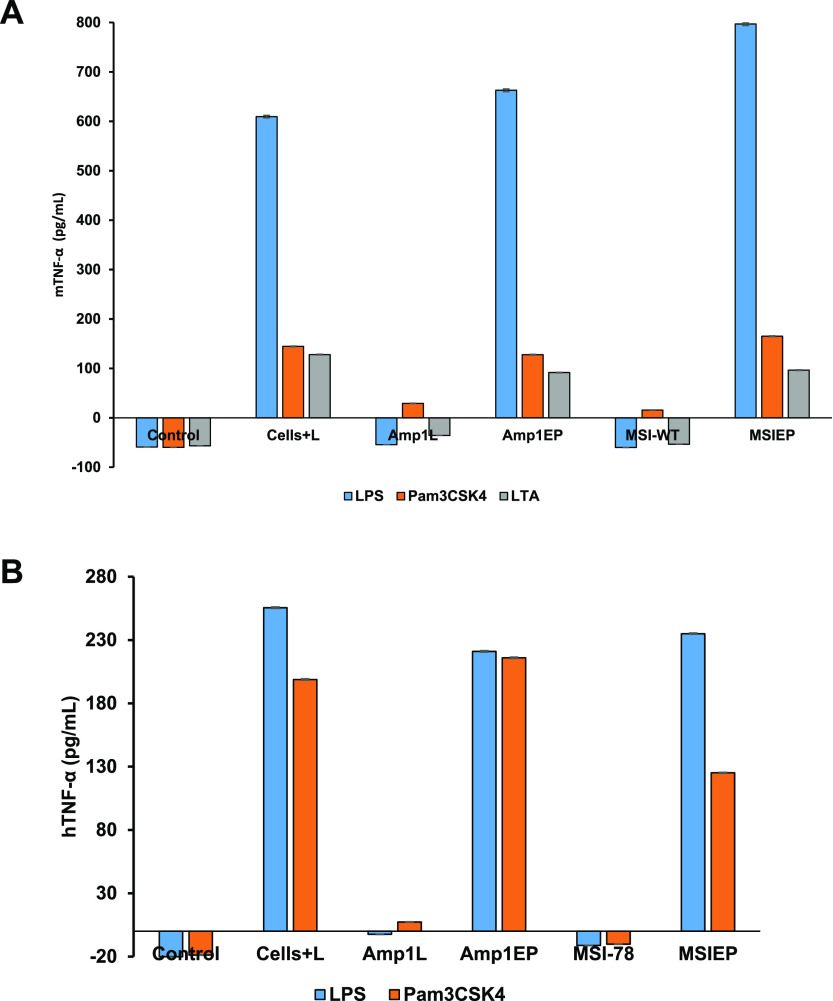
(A) Effect of the peptides on TNF-α secretion by macrophages stimulated with LPS, Pam3CSK4, and LTA (10 ng/mL) in the absence or presence of each of the different peptides at 10 μM by the ELISA assay. (B) Effect of the peptides on TNF-α secretion by human monocytic cells stimulated with LPS and Pam3CSK4 (10 ng/mL) in the absence or presence of each of the different peptides at 10 μM by the ELISA assay. Untreated cells served as the controls. Results are the mean ± SD of two independent experiments. One-way analysis of variance was used to analyze the data. Results showed a statistically significant difference (*F* = 04.95 and *p* < 0.001 and *F* = 07.32 and *p* < 0.001 for panels A and B, respectively).

In order to explore the anti-inflammatory effect of the new peptides, the levels of inflammatory cytokine TNF-α, released in response to pro-inflammatory ligands, were measured using a TNF-α enzyme-linked immunosorbent assay kit. The evaluation of the cytokine level in supernatants of THP-1 and RAW 264.7 cells after stimulation of LPS, LTA, and Pam3CSK4 (10 ng/mL) following the addition of peptides (10 μM) revealed that the levels of TNF-α decreased significantly using the parental peptides compared with the new peptides Amp1EP and MSIEP (Figure 9A,B).

## Discussion

Antimicrobial peptides are considered as novel antimicrobial candidates based on their unique antimicrobial mechanism leading to irreparable damage of bacterial membranes.^39−41^ Since antibiotic peptides are known to face a number of challenges, which include proteolytic degradation, toxicity, activation of an immune response, and others, they are being investigated using various biochemical approaches.^42,43^ Here, we showed for the first time, to our knowledge, the effect of the replacement of peptide bonds with amide bond isosteres at several positions in two parental antimicrobial peptides, Amp1L and MSI-78. This modification preserved the size, sequence, charge, and molecular weight of the parental peptides. Importantly, all the products of the complete enzymatic degradation should be amino acids. This is in contrast to many other families of AMPs composed of both amino acids and nonamino acid building blocks. Note that, whereas the modified peptides preserved practically the same MIC except against *P. aeruginosa* in the case of Amp1L, a reduced but still high antimicrobial activity was observed in the case of MSI.

Parental peptides exhibit high hemolytic activity against hRBCs and high cytotoxicity against murine macrophage (RAW 264.7) cells and human monocytic (THP-1) cells. In comparison, Amp1EP and MSIEP showed negligible hemolytic activity and reduced toxicity. Generally, it is reported that the hydrophobicity of peptides tends to increase their toxicity.^44,45^ Note that the peptides differ only in their relative hydrophobicities, which are lower in the new peptides, explaining their reduced toxicities.

Most AMPs are susceptible to protease degradation.^46^ The new peptides Amp1EP and MSIEP showed excellent stability as demonstrated in the trypsin degradation assay, while this enzyme rapidly degraded the parental peptides (Figure 5). The stability of the new peptides toward trypsin might indicate that these peptides could remain unaffected with a trypsin-like super family of serine proteases. Furthermore, we also found that both peptides incorporating isopeptide bonds were markedly more stable in blood plasma compared to the parental peptides (Table 3).

With respect to the structural analysis of peptides, the new peptides have lesser tendency to adopt defined structures when bound to LPS, in contrast to parental peptides, which have well-defined helical structures. It is noteworthy to note that the biological activity of the peptides is exclusively not defined by the secondary structure of the peptides.

The main targets of the AMPs are the cytoplasmic membranes of the bacteria in which their disruption or the formation of pore/ion channels occurs.^47^ To evaluate membrane penetrating activity, the bacterial cells were treated with the peptides at their MIC concentrations and examined by flow cytometry. The results indicated that both parental and new peptides make cells permeable on all the bacterial stains tested. However, the rate of membrane permeabization is different for each bacteria/peptide combination.

In addition to their antimicrobial activity, AMPs have been shown to exert anti-inflammatory activity and participate in the immune regulation.^48^ The inflammatory cytokine TNF-α, as a key mediator of inflammatory responses, is essential for the host response and resistance to pathogens during acute infection. All the peptides were tested to evaluate the TNF-α secretion on macrophages and human monocytic cells induced with different ligand activations. Using ELISA, we demonstrated that the Amp1EP and MSIEP showed almost no suppression of TNF-α secretion whereas the parental peptides Amp1L and MSI-78 significantly decreased TNF-α secretion on stimulated macrophages and human monocytic cells.

## Conclusions

In conclusion, here, the switch of the peptide bond to an amide bond isostere lowered the cytotoxic activity, preserved the antimicrobial activity, and significantly increased the stability of these peptides to proteolytic degradation. Importantly, the new peptides do not repress the pro-inflammatory cytokine release. Practically, we perceived less bacterial membrane perturbation observed by flow cytometry and confocal microscopy, which correlates to the structural analysis as observed by circular dichroism spectroscopy. The present findings demonstrated that the incorporation of isopeptide bonds in antimicrobial peptides could be an attractive strategy to develop them for therapeutics.

## Methods

### Peptide Synthesis and Purification

The synthesis of peptides was carried out on Rink Amide MBHA resin by using the Fmoc strategy on a Liberty Blue peptide synthesizer (CEM, Matthews, NC, United States) as reported earlier.^49^ To achieve the synthesis of new peptides Amp1EP and MSIEP, we made use of the Boc-Fmoc(Lys)-OH instead of Fmoc-Boc(Lys)-OH, which is used to make regular peptide bonds. The positions that are involved in isopeptide bond formation are K5, K9, and K13 in the case of Amp1EP and K8, K14, and K18 in the case of MSIEP. The resin-bound peptide was washed thoroughly with dry dimethylformamide (DMF) and then dry methylene chloride (DCM), dried, and cleaved. Cleavage was done by using 95% trifluoroacetic acid (TFA), 2.5% water, and 2.5% triisopropylsilane (TIS) for 120 min at room temperature. The crude peptides were washed from the resin using TFA, precipitated using cold diethyl ether, and air-dried. The purification was done by using reverse phase high performance liquid chromatography (RP-HPLC) on a C_4_ column (Grace Discovery Sciences, Columbia, MD, United States) using a linear gradient of 10–90% acetonitrile in water [both containing 0.1% TFA (v/v)] for 40 min with a flow rate of 1.8 mL/min. All the peptides were purified to >95% purity. The molecular mass of all the peptides was determined by TOF-MS.

### Antimicrobial Activity of the Peptides

The antimicrobial activity of the peptides was determined against two Gram-negative (*Pseudomonas aeruginosa* PA01 and the *Escherichia coli* K12 parental type) and two Gram-positive (*Staphylococcus aureus* and *Bacillus subtilis*) bacteria. The minimal inhibitory concentration (MIC) assays were performed as reported by Wiegand et al.,^50^ using the broth microdilution method in 96-well round-bottom microplates. Briefly, bacterial cells in mid log phase were cultured in Mueller-Hinton broth (MHB) and then diluted to 10^6^ CFU/mL. Aliquots of 50 μL of bacterial solution were added to 50 μL of MHB medium containing 2-fold serially diluted peptides with concentrations ranging from 1.56 to 50 μM. Plates were incubated at 37 °C for 24 h, and MICs were defined as the lowest concentration of peptide that prevented detectable turbidity. Cultures without peptides and uninoculated MHB were employed as positive and negative controls, respectively. The bacterial growth inhibition was evaluated by measuring the absorbance at 600 nm using a microplate autoreader (SynersyMx, Biotek).

### XTT Cell Survival Assay

The cytotoxicity of the peptides toward the murine macrophage cells (RAW 264.7) and human monocytic (THP-1) cells was assayed using the colorimetric 2,3-bis(2-methoxy-4-nitro-5-sulfophenyl)-2*H*-tetrazolium-5-carboxanilide (XTT) method. Here, 1 × 10^5^ cells per well were incubated with serially diluted peptides with concentrations ranging from 1.56 to 100 μM for 24 h at 37 °C in 5% CO_2_. Moreover, the last two columns with media served as the blank and the cells plus media of 100% survival control. After incubation, the cell viabilities were assessed by the XTT reaction solution (100 μL), and benzenesulfonic acid hydrate and *N*-methyl dibenzopyrazine methyl sulfate (mixed in a 50:1 ratio) were added for an additional 4 h incubation at 37 °C. The absorbance at 450 nm was then measured using a microplate autoreader (SynersyMx, Biotek). The percentage of cell viability was calculated relative to the 100% survival control after the blank read’s deduction.

### Hemolysis on Human Red Blood Cells (hRBCs)

Human red blood cells (hRBCs) were used to measure the hemolytic effect of the peptides by measuring the amount of hemoglobin released after treatment.^51^ Fresh human blood was obtained from healthy volunteers and processed to obtain RBCs by centrifugation at 600*g* for 5 min. The plasma was removed, and the lower layer containing RBCs was washed three times in sterile phosphate buffered saline (PBS) and centrifuged at 600*g* for 5 min. The purified hRBCs were diluted in PBS to a final concentration of 2% (v/v); then, 100 μL of the hRBC suspension was incubated with 100 μL of different concentrations (6.25 to 100 μM) of a peptide dissolved in PBS. After 1 h of incubation at 37 °C under 5% CO_2_, intact hRBCs were pelleted by centrifugation at 600*g* for 5 min and then supernatant was taken out and transferred to another 96-well plate. The sample absorbance was measured at 450 nm using a microplate autoreader (SynersyMx, Biotek). Untreated cells were used as a negative control and cells treated with 1% Triton X-100, as a positive control. The percentage of hemolysis was calculated as [(sample absorbance – negative control absorbance)/(positive control absorbance – negative control absorbance)] × 100.

### Protease Resistance Assay

Proteolysis was measured by RP-HPLC using the following parameters, as reported.^52^ Trypsin with a final concentration of 10 μg/mL was added to a solution of the peptides in phosphate buffered saline (100 μM), and the reaction was monitored over time by using reversed-phase HPLC (C_18_ reverse phase, Bio-Rad analytical column, 250 × 4 mm, 300 Å pore size, 7 μm particle size). The column was eluted in 40 min using a linear gradient of 10–90% acetonitrile in water containing 0.1% trifluoroacetic acid (v/v) at a flow rate of 0.6 mL/min.

### *In Vitro* Assay of Plasma Stability Testing

The stability of peptides in human blood plasma was measured using a literature procedure.^13,53^ Plasma was separated from red blood cells over centrifugation, frozen, and stored at −80 °C until use. Briefly, peptides were prepared as a 1 mM solution in phosphate buffered saline (pH 7.4). 20 μL of the peptide solution was diluted in 80 μL of blood plasma (20% v/v). The solution was incubated at 37 °C for different time points, and 100 μL of a mixture containing 80% acetonitrile/10% methanol/10% water was added to stop further degradation of the peptides. A cloudy solution was produced upon the addition of the stopping solution, and the sample was cooled to 4 °C for 1 h and then centrifuged at 10 000 rpm for 10 min to remove the plasma proteins as precipitate. The supernatant (50 μL) was injected onto a reversed-phase HPLC (C_18_ reverse phase, Bio-Rad analytical column, 250 × 4 mm, 300 Å pore size, 7 μm particle size). The column was eluted in 40 min using a linear gradient of 10–90% acetonitrile in water containing 0.1% trifluoroacetic acid (v/v) at a flow rate of 0.6 mL/min, and the absorbance was detected at 215 nm. The percentage of remaining peptide was determined by the decrease in chromatographic peak area.

### Structural Analysis

The secondary structure of the peptides was examined using CD on a Chirasca CD spectrometer (Applied Photophysics Ltd., Jasco, Tokyo, Japan) at 25 °C using a thermostatic quartz cuvette with a path length of 1 mm.^54^ Peptides were dissolved in phosphate buffered saline (PBS) and 5 mM HEPES (pH 7.4) to a final concentration of 50 μM in the presence or absence of 50 μM purified *P. aeruginosa* lipopolysaccharide (LPS) (Sigma-Aldrich). Each spectrum was recorded at a scanning speed of 20 s in 1 nm path length quartz cells from 190 to 250 nm.

### Evaluation of Bacterial Membrane Permeabilization by Flow Cytometry and Confocal Microscopy

The effect of the peptides on the bacterial membrane was evaluated using a Stratedigm S1000EON flow cytometer and analyzed by using CellCapTure software (San Jose, CA, USA). SYTOX green (Sigma-Aldrich) is a DNA binding dye that labels only cells with compromised membranes.^55^ SYTOX green was excited with the cyan 488 nm laser. Forward (FS) and side scatter (SSC) as well as green fluorescence emission (530/30 filter) were measured. Briefly, the experiments were done in three steps: (1) An overnight MHB culture of bacteria was diluted to a final concentration of 10^6^ cells per mL in 10 mM sodium phosphate buffer (pH 7.4). (2) SYTOX green was added to the same bacterial suspension with a final concentration of 5 μg/mL. (3) The peptides at their MIC concentrations were added to the bacteria and SYTOX green suspension. The samples were tested in the flow cytometer immediately after each step. The temporal effect of the peptide on the permeability of the bacteria membrane was measured continuously over 20 min. Next, the collected data of every 30 s was pulled together and analyzed. For each of the 30 s intervals, a histogram of the SYTOX green fluorescence levels was plotted and the percentage of bacteria with green signal over the control background level was calculated. As a control, a similar bacteria suspension with the same concentration of SYTOX green was measured for 20 min. Using the same protocol, a fluorescence confocal microscope (Olympus IX81 FV10-ASW, 60× oil objective) was used to examine the new peptides Amp1EP and MSIEP with an excitation wavelength of 488 nm.

### Measurement of TNF-α

RAW 264.7 and THP-1 cells (1 × 10^5^ cells/well) were seeded in 96-well plates and incubated for 24 h at 37 °C. Further, cells were incubated for another 4 h in the presence or absence of peptides with a final concentration of 10 μM. After incubation, lipopolysaccharide (LPS), Pam3CSK4, and lipoteichoic acid (LTA) for RAW cells and LPS and Pam3CSK4 for THP-1 cells with a final concentration of 10 ng/mL were added. The levels of pro-inflammatory cytokines (mTNF-α and hTNF-α) in the supernatants were determined after 6 h using a DuoSet ELISA kit (R&D Systems). The absorbance was measured at 450 and 540 nm using a microplate autoreader (SynersyMx, Biotek). The cytokine concentration of each well was calculated relative to the standard curve after the blank read’s deduction.
